# Intrinsic functional connectivity mediates the effect of personality traits on depressive symptoms

**DOI:** 10.1371/journal.pone.0300462

**Published:** 2024-07-10

**Authors:** Zheng Long Lee, Savannah Kiah Hui Siew, Junhong Yu

**Affiliations:** School of Social Sciences, Psychology, Nanyang Technological University, Singapore, Singapore; Imperial College London, UNITED KINGDOM

## Abstract

**Background:**

Personality traits have been proposed as risk factors for depressive symptoms. However, the neural mechanism behind these relationships is unclear. This study examined the possible mediating effect of resting-state functional connectivity networks on these relationships.

**Methods:**

Data from 153 healthy Germans were obtained from the MPI-Leipzig Mind-Brain-Body: Neuroanatomy & Connectivity Protocol database. Network-based statistics were used to identify significant functional connectivity networks that were positively and negatively associated with the personality traits of neuroticism, conscientiousness, and extraversion, with and without demographical covariates. Mediation analyses were performed for each personality trait and depressive symptoms with the significant positive and negative network strengths of the respective personality traits as mediators.

**Results:**

Neuroticism, conscientiousness, and extraversion were significantly correlated with depressive symptoms. Network-based statistics identified patterns of functional connectivity that were significantly associated with neuroticism and conscientiousness. After controlling for demographical covariates, significant conscientiousness-associated and extraversion-associated networks emerged. Mediation analysis concluded that only the neuroticism-positive network mediated the effect of neuroticism on depressive symptoms. When age and sex were controlled, the extraversion-positive network completely mediated the effect of extraversion on depressive symptoms.

**Conclusions:**

These findings revealed that patterns of intrinsic functional networks predict personality traits and suggest that the relationship between personality traits and depressive symptoms may in part be due to their common patterns of intrinsic functional networks.

## Introduction

Based on the Diagnostic and Statistical Manual of Mental Disorders, Fifth Edition (DSM-5), depressive disorders are characterised by “sad, empty, or irritable mood” along with physical and mental changes that impair people’s ability to function [1]. Depressive disorders can also be conceived as a dimension that ranges from not having depressive symptoms to minor depression to major depression [2, 3]. Some people experience subthreshold or subclinical depression, which happens when people experience some depressive symptoms but do not cross the diagnostic criteria to be diagnosed with depressive disorders [4, 5]. People with subthreshold depression are more likely to develop major depression [6–8], tend to experience impaired functioning [5, 9, 10], and face increased suicide risks [9]. It is also likely more cost-effective to treat subthreshold depression than major depression [11]. Hence, a better understanding of the aetiology of depressive symptoms can lead to earlier interventions, reducing such costs.

One factor associated with depression is personality traits. Personality traits are consistent dimensions in individuals and influence their thoughts, feelings, and behaviours [12, 13]. There are five trait dimensions: openness, conscientiousness, extraversion, agreeableness, and neuroticism [13]. High neuroticism, low conscientiousness and low extraversion are associated with depressive symptoms—the association of neuroticism is stronger than either conscientiousness or extraversion [14, 15]. Neuroticism is the tendency to experience negative affect like stress, sadness, and anger [16]. Conscientiousness is the tendency to be responsible, practise self-restraint, and strive purposefully towards goals [17]. Extraversion is the tendency to experience positive emotions, be assertive, and prefer social activities [18]. Additionally, neuroticism has been identified as a risk factor for depression in longitudinal studies [19, 20].

Studies on the mechanisms behind the relationship between personality traits and depressive symptoms have identified mediators like stress perception [21], cognitive emotion regulation strategies [22], and self-efficacy [23]. However, the neural correlates behind the relationship are unclear as there have been few studies on this. One possible neural correlate that mediates this relationship is the patterns of intrinsic functional connectivity.

Functional connectivity between a pair of regions in the brain alludes to the manner they communicate with each other [24]. When an individual attends to external stimuli or at rest, this intrinsic functional connectivity can reveal information relating to the individual, such as their personality traits. Indeed, some studies have found various patterns of functional connectivity associated with neuroticism and conscientiousness, except for extraversion, which was not as strongly or significantly related to patterns of resting-state functional connectivity (rsFC) [25–30]. Furthermore, after accounting for the effects of age and sex, rsFC patterns became more strongly predictive of conscientiousness and generally less predictive of neuroticism [25, 26]. As for extraversion, the effect of age and sex on the patterns of functional connectomes associated with extraversion has not been consistent in the literature [25, 26]. Hence, studies should conduct two models of analyses, with and without controlling for covariates, to better understand the relationships between personality traits and their patterns of intrinsic functional connectivity.

Depressive symptoms are correlated with patterns of intrinsic functional connectivity [31, 32]. Additionally, there are overlaps in the rsFC of the amygdala with other parts of the brain in separate studies investigating the relationships between the rsFC of amygdala and personality traits and between amygdala rsFC and depression—neuroticism and depression are correlated with amygdala rsFC with the temporal poles, insula, and precuneus; and extraversion and depression are correlated with amygdala rsFC with insula, putamen, and temporal pole [33, 34]. These studies suggest that certain patterns of intrinsic functional networks associated with personality traits might mediate the relationship between personality traits and depressive symptoms.

This study aims to examine whether patterns of intrinsic functional connectivity that are significantly associated with personality traits mediate the effect of personality traits on depressive symptoms. We hypothesise that neuroticism, conscientiousness, and extraversion predict depressive symptoms. Additionally, we hypothesise that various patterns of intrinsic functional connectivity networks are significantly associated with different personality traits, although the inclusion of demographical covariates may alter these relationships. Finally, we tested brain mediation hypotheses to determine whether patterns of intrinsic functional connectivity networks that predict personality traits would mediate the relationship between that personality trait and depressive symptoms.

## Materials and methods

### Study participants

Data from 204 healthy German participants with the Neuroanatomy & Connectivity Protocol (N&C) data in the MPI-Leipzig Mind-Brain-Body (MPILMBB) database were used [35, 36]. The dataset is in the public domain. The MRI dataset and the behavioural data used in this study were accessed at https://openneuro.org/datasets/ds000221/versions/1.0.0 and http://nitrc.org/projects/mpilmbb/, respectively. The data was accessed on 8 Jan 2022, and we did not have access to any identifying information during or after data collection. A total of 157 participants were left after excluding participants who (1) did not take the NEO Personality Inventory-Revised (NEO PI-R), (2) did not undergo their resting state fMRI (rs-fMRI) scans, or (3) had excessive head motion during their rs-fMRI scans. 75 of the participants are female (47.8%). The participants’ age was grouped into 5-year bins (e.g., 20 to 25, 25 to 30, etc.), and the median 5-year age bins is between 25 and 30. For ease of analysis, participants’ sexes were recoded into integers (male = 0, female = 1), while the 5-year age bins were recoded into integers (20 to 25 = 1, 25 to 30 = 2).

The study protocol was approved by the ethics committee at the medical faculty of the University of Leipzig (097/15-ff), and all participants provided written informed consent.

### Procedure

The German version of the NEO Personality Inventory-Revised (NEO PI-R) was used to measure the five personality traits: openness, conscientiousness, extraversion, agreeableness, and neuroticism [37, 38]. NEO PI-R contains 240 items with 48 items for each trait. Each item is scored on a 5-point Likert scale, ranging from 1 (Strongly Disagree) to 5 (Strongly Agree). Participants with higher scores on a personality trait are more likely to exhibit the trait. As noted by Mendes et al. [35] in the N&C of the MPILMBB database, there was a repeat measurement of item 71 (“I am seldom sad or depressed”) and they omitted items 46 and 83. Hence, items 46 and 83 were unaccounted for in creating the subscale N3 and N5 summary scores, respectively.

The German version of the Beck Depression Inventory-II (BDI-II) was used to measure the severity of depressive symptoms in the participants [39, 40]. It contains 21 multiple-choice items and is scored on a 4-point Likert scale from 0 to 3. The scores range from 0 to 63, and participants with higher scores experience more severe depressive symptoms. The range of scores for these participants is from 0 to 25.

### MRI acquisition

The structural and rs-fMRI scans were also taken from the MPI-Leipzig Mind-Brain-Body (MPILMBB) database [35]. The scans were obtained using a 3T scanner (MAGNETOM Verio, Siemens Healthcare, Erlangen, Germany) equipped with a 32-channel head coil.

The structural scans were acquired using a 3D MP2RAGE sequence [41]. The parameters include repetition time (TR) = 5000 ms; echo time (TE) = 2.92 ms, first inverse time (TI1) = 700 ms; second inverse time (TI2) = 2500 ms; flip angle 1 = 4°; flip angle 2 = 5°, bandwidth = 240 Hz/Px; voxel size = 1.0 mm isotropic. The MP2RAGE sequence produces two images at different inversion times (inv1 and inv2). These images are a quantitative T1 map (t1map) and a uniform T1-weighted image (t1w), respectively.

The rs-fMRI scans were acquired using a gradient-echo echo planar imaging (GE-EPI) with multiband acceleration. Further parameters include TR = 1400 ms; TE = 39.4 ms; flip angle = 69°; echo spacing = 0.67 ms; imaging matrix = 88 × 88, 64 slices with 2.3 mm thickness; bandwidth = 1776 Hz/Px; voxel size = 2.3 mm isotropic. During their rs-fMRI scans, participants were instructed to remain awake with open eyes and to focus on a fixation cross.

### Image pre-processing

The T1 structural images are pre-processed with FreeSurfer 7.2.0 using the default recon-all options. This process involves the removal of non-brain tissue using a hybrid watershed/surface deformation procedure [42], automated Talairach transformation, segmentation of the subcortical white matter and deep grey matter volumetric structures (including the hippocampus, amygdala, caudate, putamen, ventricles) [43, 44], intensity normalisation [45], tessellation of the grey matter white matter boundary, automated topology correction [46, 47], and surface deformation following intensity gradients to optimally place the grey/white and grey/cerebrospinal fluid borders at the location with the greatest shift in intensity, which defines the transition from one tissue class to another [48, 49].

The resting state fMRI volumes were pre-processed using fMRIPrep 20.2.5 [50]. Functional data were slice time corrected using 3dTshift from AFNI [51] and motion corrected using MCFLIRT [52]. This process was followed by co-registration to the corresponding T1w using boundary-based registration [53] with 9 degrees of freedom, using bbregister from freesurfer. Motion correcting transformations, BOLD-to-T1w transformation and T1w-to-template (MNI) warp were concatenated and applied in a single step using antsApplyTransforms employing Lanczos interpolation.

Subsequently, these pre-processed volumes were denoised by regressing out 6 motion parameters, the average signal of white matter and cerebrospinal fluid masks, global signal, and their derivatives, as well as cosines covering slow time drift frequency band using the load_confounds package (https://github.com/SIMEXP/load_confounds) in python. Scrubbing was carried out to further remove the effects of excessive head motion [54]. The volumes are then smoothed using a 5mm FWHM kernel and subjected to a 0.1Hz low-pass filter. Finally, the brainnetome atlas [55] was used to parcellate the whole brain into 246 anatomical regions corresponding to the nodes of the network. Participants with excessive head motion, defined by having more than 20% of their rsfMRI volumes above the high motion cutoff (relative RMS > 0.25), are excluded from the analyses.

### Statistical analyses

In this study, two sets of statistical analyses were conducted: a baseline model whereby age and sex are not controlled, and a covariate model whereby age and sex are controlled.

For the baseline model, three separate network-based analyses were conducted on the rsFC matrices to acquire edges that correlated with the three personality traits (neuroticism, conscientiousness, and extraversion) using linear models with 1000 permutations and a threshold of p < .001. Significant edges that correlated positively with that personality trait are positive edges, while those that correlated negatively are negative edges. The functional connectivity (FC) networks, made of some of these edges, are significantly associated with the personality trait if the probabilities for the networks to be formed were p < .05. If the FC networks are significant, the positive and negative connectivity scores for that personality traits are calculated for each participant using the connectivity strength of the positive and negative edges of the FC networks. Higher scores indicate higher connectivity strength.

Network-based statistics, as implemented in the R package ‘NBR’ [56], were used to identify networks of rsFC edges that are significantly associated with the different personality traits, with and without controlling for age and sex. Selection thresholds were set at *p* < .001 and *p* < .05 at the edge and network levels, respectively. Statistical significance at the network level was determined by testing its network strength against a null-distribution generated by 1000 nonparametric permutations. For each of the personality traits, the significant edges that survived both levels of thresholds are grouped into positive and negative networks, corresponding to their positive and negative associations with the trait. Then, the positive and negative network strengths were calculated by summing up these positive and negative edges in their respective groups. To test the brain mediation hypotheses, these network strengths were entered into the subsequent mediation analyses as mediators in the relationship between personality traits and depressive symptoms. The statistical significance of the indirect effect was assessed using the bootstrapping approach with 5000 bootstraps as implemented in the R package ‘psych’ [57].

For the covariate model, the same procedures in the baseline model were repeated with the exception that the network-based analysis was conducted on the rsFC matrices to obtain edges that correlate with the three personality traits while controlling for the covariates of 5-year bins and sex.

## Results

### Participant data

Table 1 contains the descriptive statistics of the participants, and Table 2 shows the correlation between personality traits and depressive symptoms. Since only neuroticism, conscientiousness and extraversion are correlated with depressive symptoms, no further analyses were conducted for agreeableness and openness to experience.

**Table 1:** Descriptive statistics of participants.

	Age Bin								
	20–25	25–30	30–35	35–40	40–45	60–65	65–70	70–75	75–80
**Number of Participants**	56	58	14	6	1	4	11	4	3
**Sex**									
Male	29	35	9	2	0	2	3	2	1
Female	28	23	5	4	1	2	8	2	2
**BDI-II**	6.19	5.84	5.07	8.33	0	4.25	6.56	2.75	3.33
**NEO PI-R**									
Neuroticism	88.14	84.6	80.79	93.33	92	65.5	75.33	62.75	86.67
Extraversion	119.64	119.47	115.43	108	116	119	113.33	114.25	108.67
Openness to Experience	122.91	121.71	124.93	148	145	131	110.67	108.75	100.33
Agreeableness	128.21	128	135.29	125.17	133	126.25	133.33	136.5	127.67
Conscientiousness	114.79	119.03	105.57	110.5	121	124.5	129.44	145.25	122.33

BDI-II, Beck Depression Inventar-II, measuring depressive symptoms; NEO PI-R, NEO Personality Inventory-Revised. Mean scores of BDI-II and NEO PI-R are presented

**Table 2:** Correlations between personality traits and depressive symptoms.

	NEO PI-R				
	Neuroticism	Conscientiousness	Extraversion	Openness to Experience	Agreeableness
**BDI-II**	0.57***	-0.27***	-0.21**	-0.12	-0.13

BDI-II, Beck Depression Inventar-II, measuring depressive symptoms; NEO PI-R, NEO Personality Inventory-Revised. Correlations between BDI-II and the respective personality traits are shown.

*p < .05;

**p < .01;

***p < .001

### Network-based analyses

**Fig 1:**
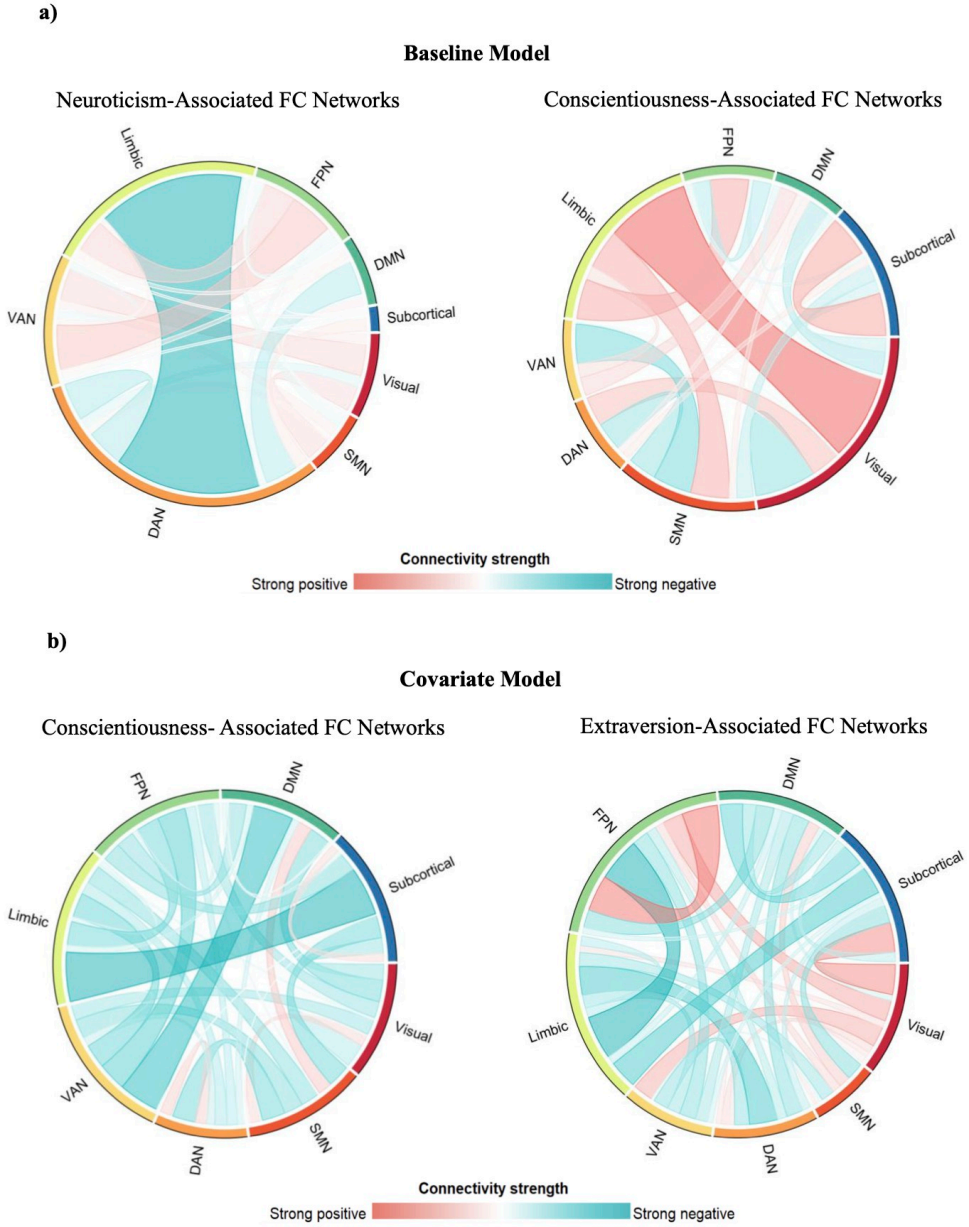
Functional connectivity networks. Chord diagrams illustrate certain patterns of FC networks which are significantly predictive of the personality traits a) without controlling for age and sex and b) after controlling for age and sex. A positive strength between a pair of networks would suggest that stronger positive connectivity between them is associated with higher scores on the personality trait. DAN = dorsal attention network. VAN = ventral attention network. FPN = frontoparietal network. SMN = somatomotor network. DMN = default mode network.

In the baseline model, for which covariates were not controlled, only the neuroticism-associated networks (p = .005) and conscientiousness-associated networks (p = .016) were significant. The extraversion-related FC network was not significant (p = .152). In the covariate model whereby age and sex were controlled, only the conscientiousness-related FC networks (p = .047) and extraversion-related FC networks (p = .046) were significant, while neuroticism-related FC networks were not (p = .061). The chord diagrams of the networks in the baseline and covariate models are shown in Fig 1. The significant edges were grouped into the seven resting-state brain networks according to the parcellation by Yeo et al. [58] and a subcortical region. The positive and negative connectivity patterns formed the positive and negative FC network of the personality traits, respectively.

According to these chord diagrams in Fig 1, the limbic-dorsal attention network (DAN) connectivity was strongly and negatively related to neuroticism. The connectivity with the strongest positive strength in the conscientiousness-associated FC networks was the limbic-visual connectivity. For the conscientiousness-associated FC networks in the covariate model, the ventral attention network (VAN)- default mode network (DMN) connectivity was strongly and negatively correlated with conscientiousness. For the extraversion-associated FC networks in the covariate model, the frontoparietal network (FPN)-FPN connectivity was most strongly positively associated with extraversion.

### Mediation analyses

Only the neuroticism-positive FC network in the baseline model and the extraversion-positive FC network in the covariate model mediated the effect of that personality trait on depressive symptoms. The neuroticism-positive FC network in the baseline model partially mediated this effect, whereas the extraversion-positive FC network completely mediated the effect of extraversion on depressive symptoms in the covariate model. This information is shown in the Tables 3–6.

**Table 3:** Estimates of mediation effect of neuroticism-associated FC networks in baseline model on the effect of neuroticism on depressive symptoms.

FC Network	Effects	Estimates	95% CI Lower	95% CI Higher
**Positive**	Indirect Effect	0.1*	0.02	0.2
	Direct Effect	0.46*	0.3	0.62
	Total Effect	0.57*	0.43	0.71
**Negative**	Indirect Effect	0.03	-0.06	0.12
	Direct Effect	0.54*	0.38	0.70
	Total Effect	0.57*	0.43	0.71

*p < .05.

**Table 4:** Estimates of mediation effect of conscientiousness-associated FC networks in baseline model on the effect of conscientiousness on depressive symptoms.

FC Network	Effects	Estimates	95% CI Lower	95% CI Higher
**Positive**	Indirect Effect	0.11	0	0.21
	Direct Effect	-0.37*	-0.57	-0.17
	Total Effect	-0.27*	-0.43	-0.11
**Negative**	Indirect Effect	0.01	-0.11	0.13
	Direct Effect	-0.28*	-0.48	-0.08
	Total Effect	-0.27*	-0.43	-0.11

*p < .05.

**Table 5:** Estimates of mediation effect of conscientiousness-associated FC networks in covariate model on the effect of conscientiousness on depressive symptoms.

FC Network	Effects	Estimates	95% CI Lower	95% CI Higher
**Positive**	Indirect Effect	-0.05	-0.25	0.14
	Direct Effect	-0.21	-0.46	0.04
	Total Effect	-0.27*	-0.43	-0.11
**Negative**	Indirect Effect	-0.07	-0.15	0.02
	Direct Effect	-0.2	-0.42	0.02
	Total Effect	-0.27*	-0.43	-0.11

*p < .05.

**Table 6:** Estimates of mediation effect of extraversion-associated FC networks in covariate model on the effect of conscientiousness on depressive symptoms.

FC Network	Effects	Estimates	95% CI Lower	95% CI Higher
**Positive**	Indirect Effect	-0.18*	-0.3	-0.05
	Direct Effect	-0.03	-0.23	0.17
	Total Effect	-0.21*	-0.37	-0.05
**Negative**	Indirect Effect	-0.07	-0.15	0.02
	Direct Effect	-0.14	-0.32	0.04
	Total Effect	-0.21*	-0.37	-0.05

*p < .05.

## Discussion

As hypothesised, our study supports past findings that neuroticism, conscientiousness, and extraversion significantly predicted depressive symptoms, whereas openness to experience and agreeableness did not [14, 15]. While a recent study found that neuroticism, extraversion and openness to experience were associated with General Health Questionnaire-12B (GHQ-12B) [59], the difference in findings might be due to GHQ-12B evaluating both depression and anxiety rather than depressive symptoms. This study found that in the baseline model, certain patterns of rsFCs predicted neuroticism and conscientiousness. However, only the neuroticism-positive FC network mediated the effect of the personality trait on depressive symptoms. For the covariate model, certain patterns of rsFCs also predicted conscientiousness and extraversion. However, only the extraversion-positive FC network mediated the effect of the personality trait on depressive symptoms.

The finding that certain patterns of FC networks were associated with neuroticism in the baseline model and not in the covariate model supported previous studies that found that the correlation between neuroticism and patterns of intrinsic functional connectivity decreased or became insignificant after controlling for age and sex [25, 26]. This suggests that the correlation between neuroticism and certain patterns of intrinsic functional connectivity might be accounted for by age and sex. This influence might be due to neuroticism being generally higher in females compared to males [60, 61] and the presence of sex differences in resting state functional connectivity [62, 63]. Additionally, neuroticism has been associated with age [64–66].

In the baseline model, the decreased connectivity within the DAN was negatively associated with neuroticism. This had been previously reported in a functional connectivity study [29]. DAN exerts a top-down and goal-directed influence on visuospatial attention [67–69]. A negative correlation suggests that people higher on neuroticism might be worse at controlling their attention to external stimuli and focusing on negative stimuli. This has been found in studies whereby people high on neuroticism show attentional bias for negative stimuli [70, 71].

Additionally, the limbic-DAN connectivity is the strongest in the neuroticism-negative FC network. This connectivity might be related to emotional regulation. Neuroticism is associated with a decreased usage of cognitive reappraisal [72, 73], which is an adaptive emotional regulation method [74]. Cognitive appraisal involves reinterpreting an emotional situation to modify its emotional impact [72, 75]. The limbic network is involved in emotional processing and social cognition [76–78], and the DAN is involved in top-down attentional control that might be implicated in cognitive appraisal. Therefore, the weaker limbic-DAN connectivity might suggest that people high in neuroticism have a weaker ability to use emotional regulation strategies that utilise attention to regulate negative emotions. In this study, the neuroticism-negative FC network was not a significant mediator.

In the baseline model, the neuroticism-positive FC network mediates the effect of neuroticism on depressive symptoms. The increased VAN-FPN connectivity associated with neuroticism has the strongest positive connectivity. A meta-analysis comparing rsFC of individuals with MDD and healthy controls found that there were reports of both increased and decreased connectivity between areas of VAN and FPN across studies [32]. Although results vary, an increased connectivity might explain why individuals high on neuroticism are more vulnerable to depressive symptoms. VAN and FPN have been found to be activated as the main neural networks in tasks involving response inhibition [79]. Response inhibition is the ability to restrain one’s actions that are unsuitable for the context [80, 81]. Depressive symptoms have also been associated with deficits in response inhibition [82–84]. These suggest that the increased VAN-FPN connectivity associated with neuroticism might lead to altered response inhibition in people high on neuroticism, and the altered response inhibition might predispose them to depressive symptoms.

Moreover, the mediation effect of neuroticism on depressive symptoms is partial. This suggests that other mechanisms besides the neuroticism-positive FC network can account for how people high in neuroticism tend to experience depressive symptoms. As mentioned above, the other mechanisms are likely cognitive emotion regulation strategies, stress perception and self-efficacy. Other mediators include social inhibition, rumination, and worry [85, 86].

Our results suggest that conscientiousness is significantly correlated with certain patterns of rsFC and support past studies [25, 29]. In the baseline model, the limbic-visual connectivity is the strongest in the conscientiousness-associated network. We propose that it might be related to people high in conscientiousness being better at motivating themselves to complete tasks. The visual network contains much of the occipital cortex [58], which is involved in visual processing and visual imagery [87–89]. The limbic network is involved in emotional processing, as described above and regulating motivation [90, 91]. Hence, the increased limbic-visual connectivity associated with conscientiousness might suggest that the mental imagery of goals in people high in conscientiousness leads to stronger motivation to achieve them. This is supported by conscientiousness being associated with striving purposefully towards goals and being hardworking [17].

In the covariate model, there is a strong DMN-VAN connectivity in the conscientiousness-negative network. During a distraction suppression task, a positive DMN-VAN connectivity was associated with a decreased ability to suppress distractions [92]. Thus, the strong negative DMN-VAN connectivity pattern associated with conscientiousness might be related to increased distraction suppression. This is supported by how people high in conscientiousness are good at suppressing distractions and prioritising goals [93].

However, these conscientiousness-associated FC networks in both models do not mediate the effect of conscientiousness on depressive symptoms, unlike our hypothesis. This might be because this study focused on people with only mild depressive symptoms, and a mediating effect might be found if participants with more severe depressive symptoms are included. Other factors found to mediate the effect of conscientiousness on depressive disorders include impairment in functioning, sleep latency and daytime dysfunction [94].

Extraversion has been found to be unrelated to patterns of FC networks in the whole-brain analysis approach [25, 30]. Our study supports this finding because no significant association between extraversion and patterns of FC networks are found in the baseline model. However, after controlling for age and sex, significant associations between patterns of FC networks and extraversion are found. This supports the study by Hsu et al. [26] that extraversion was significantly correlated with certain patterns of FC networks in a whole-brain analysis when age and sex are controlled.

The extraversion-positive FC network also completely mediates the effect of extraversion on depressive symptoms and could be how extraversion relates to depressive symptoms. The increased within-FPN connectivity associated with extraversion had the strongest positive connectivity strength. This area is involved in cognitive control and the coping of task requirements by modifying their functional connectivity with the rest of the brain [95, 96]. Additionally, FPN also participates in emotional regulation. A study found that within-FPN connectivity is negatively related to the tendency to use expressive suppression to regulate emotions [97]. Expressive suppression is one of two types of emotional regulation strategies described by Gross and John [72] that involves the inhibition of emotional displays and is positively related to depressive symptoms [72, 98, 99]. Additionally, decreased within-FPN connectivity is also associated with MDD [32, 100]. These suggest that the increased within-FPN network associated with extraversion might explain why people high in extraversion are less prone to depressive symptoms since they might be less likely to use expressive suppression and might be better at managing their emotions and task demands.

This study suggests that personality traits have their unique neural fingerprints and that these patterns of intrinsic functional connectivity can be used in the objective assessments of personality traits. This also suggests how people with certain personality traits are more vulnerable to depressive symptoms. Additionally, the results highlighted the importance of accounting for age and sex when calculating the edges associated with personality traits, especially for extraversion. This study also suggests the possibility of using interventions that influence brain functional connectivity, like meditation and neurofeedback training [101–103], for people with depressive symptoms. More importantly, these interventions can be tailored according to people’s personality traits.

This study has some limitations that need to be considered. Firstly, this study is correlational. The direction and causality of the relationship among the personality traits, depressive symptoms and the patterns of FC networks associated with the respective personality traits cannot be exactly determined. The pattern of FC networks may contribute to both traits and depressive symptoms. Future studies should investigate the causality of this relationship. Secondly, the participants involved in this study do not include individuals with depressive disorders. Additionally, the BDI-II scores of the participants in this study only range from 0 to 25, whereas the full range is 0 to 65. Hence, the extrapolation of this result to people with depressive disorders and the generalisation of results to people with more severe depressive symptoms might not be appropriate. However, this study does suggest the likelihood that such a relationship might be found in people with depressive disorders and people with more severe depressive symptoms. Future replications can be conducted with participants with depressive disorders or with more severe depressive symptoms. Another limitation is that the personality traits were assessed only via self-report, which might have diminished its validity. According to the self-other knowledge asymmetry theory, self-reports are more accurate than informant ratings for traits that are harder to observe, like neuroticism, while ratings by informants should be more accurate than self-ratings for socially desirable traits like openness to experience, conscientiousness and agreeableness because of self-biases [104, 105]. While findings have been mixed, informant ratings likely have unique predictive validity over self-ratings [104, 106]. Hence, future research can use a composite measure to assess personality traits. Another limitation includes using 5-year age bins instead of the exact age, which can introduce variability to the analysis. Lastly, the participants are all German, and a replication with a more diverse participant sample can help us understand the generalisability of this conclusion to a more diverse population.

## Conclusions

Our findings suggest that patterns of intrinsic functional networks predict personality traits and that the relationship between personality traits and depressive symptoms may be explained by the patterns of intrinsic functional networks of those personality traits.
